# Does folic acid supplementation have a positive effect on improving memory? A systematic review and meta-analysis of randomized controlled trials

**DOI:** 10.3389/fnagi.2022.966933

**Published:** 2022-11-28

**Authors:** Camellia Akhgarjand, Sara Ebrahimi Mousavi, Zahra Kalantar, Amir Bagheri, Hossein Imani, Hamid Rezvani, Mahmoud Dehghani Ghorbi, Zahra Vahabi

**Affiliations:** ^1^Department of Clinical Nutrition, School of Nutritional Sciences and Dietetics, Tehran University of Medical Sciences, Tehran, Iran; ^2^Department of Cellular and Molecular Nutrition, School of Nutrition Science and Dietetics, Tehran University of Medical Sciences, Tehran, Iran; ^3^Department of Community Nutrition, School of Nutritional Sciences and Dietetics, Tehran University of Medical Sciences, Tehran, Iran; ^4^Hemato-Oncology Ward, Taleghani Hospital, Shahid Beheshti University of Medical Sciences, Tehran, Iran; ^5^Hemato-Oncology Ward, Imam Hossein Hospital, Shahid Beheshti University of Medical Science, Tehran, Iran; ^6^Cognitive Neurology and Neuropsychiatry Division, Department of Psychiatry, Roozbeh Hospital, Tehran University of Medical Sciences, Tehran, Iran; ^7^Department of Geriatric, Ziaeian Hospital, Tehran University of Medical Sciences, Tehran, Iran

**Keywords:** folic acid, cognition, memory, psychological testing, oral supplementation

## Abstract

**Introduction:**

The results of randomized controlled trials (RCTs) on the effect of folic acid supplementation on memory status due to various heterogeneity, dosage, duration, and cognitive function assessments were inconclusive. Therefore, we have performed a systematic review and meta-analysis to investigate the effect of folic acid supplementation on memory in RCTs.

**Method:**

Comprehensive computerized systematic searches were conducted throughout Scopus, PubMed/Medline, and Google Scholar from inception until February 2022 to investigate the effect of folic acid supplementation memory levels in RCTs. The standardized mean difference (SMD) and 95% confidence interval (CIs) were used to estimate the overall effect size using random-effects meta-analyses.

**Results:**

The overall results of nine trials with 641 participants, revealed that folic acid supplementation did not significantly change memory score compared to placebo (SMD: 0.12; 95% CI: −0.17, 0.40, *p* = 0.418; *I*^2^ = 62.6%). However, subgroup analyses showed that supplementation with folic acid had favorable effects on memory levels considering the following conditions: (1) doses lower than 1 mg/day, (2) treatment lasting more than 6 months, (3) conducted in eastern countries, and (4) in participants equal to or older than 70 years old. The dose-response analysis suggested a significant favorable effect on memory status at doses of 6–11 mg/d and a significant decline at doses of 17–20 mg/d.

**Discussion:**

Although we did not find a significant effect of folic acid supplementation on memory, there were some suggestions of beneficial effects in the subgroup analyses.

## Introduction

Memory is a fragmentary process and its subtypes are localized to different anatomical sites. The aging process has an impact on decreasing memories-conscious, specific memories of episodes and events, in addition to semantic information (Small, 2001). Neurodegenerative disorders, Exposure to toxic elements, infectious disease, oxidative stress, changes in cerebrovascular supply, and gestational hormonal levels can cause memory decline (Small, 2001; Albert, 2002). Both external factors [drugs such as erythropoietin (EPO), imidazobenzodiazepine (IBZD), and histamine] and internal factors (such as a diet rich in omega 3 and consuming foods containing probiotics) can improve memory (Schneider et al., 2020). As the population ages, the incidence of disease-related memory decline is increasing and now becoming a worldwide health problem (Petersen et al., 2001; Ferri et al., 2005; Li et al., 2018). Progressive memory loss is generally accepted as a sign of Mild Cognitive Impairment (MCI) and Alzheimer’s disease (AD) in most patients. There is no certain cure for disease-related memory decline such as Alzheimer’s Disease, Vascular Dementia (VD), and MCI therefore, focusing on the management of the risk factors will be the best strategy to take (Morris et al., 2001; Dam et al., 2017). Although the main mechanism of memory decline is still uncertain, inflammatory states alongside homocysteine levels are presented as two possible pathological mechanisms (Bell et al., 1992; Rosenberg, 2001; Gezen-Ak et al., 2013; Ilkjaer et al., 2014). Due to the toxic role of homocysteine on glutathione peroxidase activity and antioxidant vitamin levels, neurons become more sensitive to oxidative stress (Chen et al., 2016). In addition, some experimental studies implied folic acid could improve memory by reducing homocysteine levels (de Lau et al., 2007; Shooshtari et al., 2012). Folate is an essential nutrient that naturally occurs in reduced form in foods, folate is present in an oxidized synthetic form (folic acid) in dietary supplements and fortified foods (Medicine et al., 1998). Folic acid was investigated in our study. folic acid deficiency is one of the common conditions in the elderly (Moretti et al., 2017). Folic acid has crucial functions in the nervous system and its necessity becomes more important in individuals in their later life. It has been estimated that low serum folic acid levels increase the risk of memory decline by about 90% (Ravaglia et al., 2005; Dolatabadi et al., 2012; Ebara, 2017). Folic acid deficiency causes impaired vitamin B12 metabolism, which leads to an inflammatory state (Das, 2008). Moreover, low serum folic acid concentration increases homocysteine levels, which is mentioned as a risk factor for cognitive decline (Rosenberg, 2001; Smith, 2008). Several studies investigated the role of folic acid supplementation on memory impairment through homocysteine levels reduction (Nilsson et al., 2001; Sommer et al., 2003; Dam et al., 2017). It was reported in observational studies that the risk of memory disorder in individuals with lower levels of folic acid is much higher than that in people who are in the normal range of folic acid (Ebara, 2017). In addition, the results of randomized controlled trials (RCTs) due to various heterogeneity, dosage, duration, and cognitive function assessments are not conclusive (Eussen et al., 2006; Durga et al., 2007; Aisen et al., 2008; Higgins et al., 2019). It was reported in a trial that folic acid supplementation could not make any improvements on cognitive function and memory (Sommer et al., 2003). Moreover, another study illustrated that memory traits respond to folic acid regarded to the initial stage of folic acid deficiency (Fioravanti et al., 1997). On the contrary, Loria-Kohen et al. (2013) showed significant changes in cognitive scores after 6 months of folic acid therapy. Overall, these disagreements intrigued us to investigate through a meta-analysis whether folic acid supplementation affects memory impairment or not.

## Method

The preferred reporting items for systematic reviews and meta-analyses (PRISMA) checklist was used to report this systematic review and meta-analysis.

### Search strategy

We performed comprehensive computerized systematic searches throughout PubMed/Medline, Scopus, and Google Scholar from inception until February 2022. To find relevant articles, the following keywords containing MESH (Medical Subject Headings) and non-MESH terms were applied: (“Cognitions” OR “Dementia” OR “Memory” OR “Alzheimer’s disease” OR “Cognitive Function”) AND (“Folic acid” OR “Vitamin M” OR “Vitamin B9”) AND (“Single-Blind Method” OR “Double-Blind Method” OR “Clinical Trials as Topic” OR RCT). More details on search strategy were provided in a Supplementary Table 1. No language restriction was applied. We also manually checked all reference lists of related articles to avoid missing any relevant papers. PubMed’s e-mail alert service was activated as a tool to find any new additional articles that may have looked on this after our initial search.

### Selection criteria

The population, intervention, comparison, outcome, and study design (PICOS) criteria used for the present meta-analysis are presented in Table 1. We included studies in this meta-analysis that met the following criteria: (1) RCTs with either parallel or crossover design; (2) were carried out on adult people (≥18 years old); (3) RCTs that assessed the effects of oral folic acid supplementation on memory compared with the placebo; (4) RCTs which reported sufficient information on pre-and post-supplementation for memory in both intervention and placebo groups.

**TABLE 1:** The population, intervention, comparison, outcome, study design (PICO) criteria.

Criteria	Description
Population	Adults (aged ≥18 years)
Intervention	Folic acid supplement
Comparison	Placebo or no intervention
Outcome	Changes in memory score
Study design	Randomized controlled trials

Articles were excluded if (1) were performed on children and pregnant women; (2) had a non-RCT design including observational studies, *in vitro* studies, letters, conference papers, dissertations, patents, and protocol studies; (3) did not have any placebo group to compare the results with the intervention; (4) contained incomplete information about the selected outcomes in the intervention or placebo groups.

### Data extraction

Two independent authors (CA and AB) extracted the following information from all the qualified papers: first author’s name, year of publication, duration of intervention, mean age and gender of participants, study location, design of the study (parallel or crossover), participant’s health condition, details of the intervention including dose, number of cases and controls, and mean ± standard deviation and/or changes of the outcomes including memory tests before and after supplementation in both intervention and control groups. Furthermore, if included trials provided effect size for different periods of time, the longest intervention was extracted.

### Quality assessment

#### Assessment of risk of bias

Two authors (CA and ZK) assessed the risk of bias of the included trials by using the Cochrane Collaboration Risk of Bias tool based on random sequence generation, concealed allocation, blinding of participants, investigator and outcome assessment, incomplete outcome data, selective reporting, and other biases (Higgins et al., 2011). Trials were classified as good quality (low risk of bias for all domains), fair (high risk of bias for at most 1 item), or poor (high risk of bias for >2 items). With regard to study quality, three studies had good quality (Connelly et al., 2008; Ma et al., 2019; Bai et al., 2021), four trials were fair (Fioravanti et al., 1997; Sommer et al., 2003; Eussen et al., 2006; Pathansali et al., 2006) and two articles had poor quality (Loria-Kohen et al., 2013; Chen et al., 2016). The allocation concealment and blinding (outcome assessment) were common biases. Only two papers provided information about all domains (Connelly et al., 2008; Bai et al., 2021). The detailed results for assessment of the risk of bias are summarized in Supplementary Table 2.

### Statistical analysis

The mean change and standard deviation of memory scores were used to calculate pooled effect size. The overall effect size was estimated using random-effects meta-analyses, and the standardized mean difference (SMD) and 95% confidence interval (CIs) were used. We applied the following formula to calculate mean change (SD) for studies that did not report it: SD_*change*_ = square root [(SD_*baseline*_)^2^ + (SD_*final*_)^2^−(2R × SD_*baseline*_ × SD_*final*_)], and mean change = final values–baseline values. The best correlation coefficient (R) was determined by examining studies that reported mean (SD) changes (Borenstein et al., 2021). Moreover, we extracted numerical estimates from graphs using Get Data Graph Digitizer version 2.24 (Fedorov, 2002).

Subgroup analyses were done on the basis of predefined variables including dosage of supplementation, mean age, sample size, duration of intervention, and study location and health status of participants. To account for the obvious heterogeneity in study designs, we applied random-effects models. The I-squared index was used to assess study heterogeneity. If the I^2^ was greater than 50%, there was heterogeneity between the included trials (Mousavi et al., 2022). A sensitivity analysis was used to evaluate the potential bias and robustness of the overall effect estimate (Mousavi et al., 2021). To determine the presence of publication bias, Egger’s regression test as well as funnel plot were used. Additionally, non-linear associations were investigated using fractional polynomial models (polynomials) (Jie et al., 2018). Stata software (Stata Crop, College Station, TX, USA) version 14 was used for all statistical analyses. A significance level of *p*-values <0.05 was considered statistically significant.

### Grading the evidence

We used the Grading of Recommendations Assessment, Development and Evaluation (GRADE) approach to rate certainty of evidence (Schunemann, 2008).

## Results

### Study selection

**FIGURE 1:**
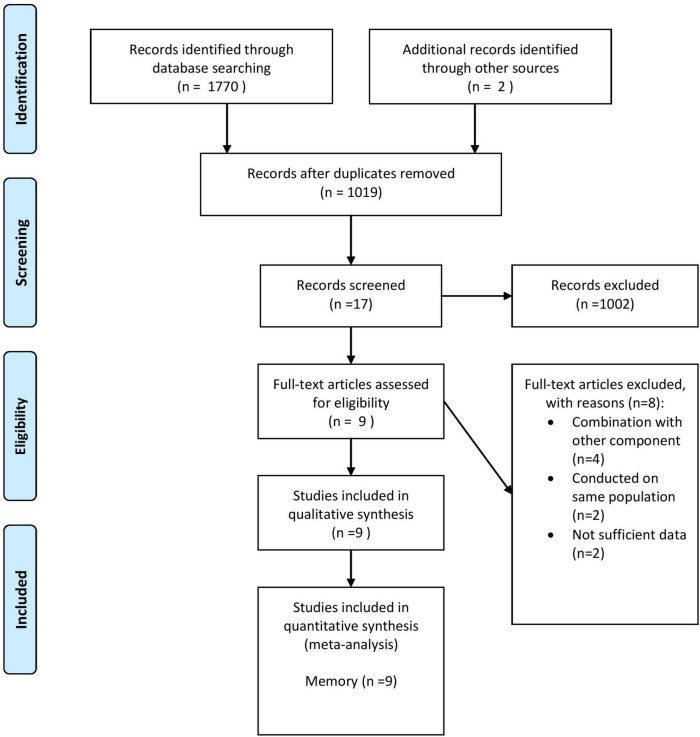
Flow diagram of study selection. Adopted from Moher et al. (2009).

According to the initial database search, 1,772 publications (436 from PubMed, 1,333 from Scopus, and 2 from other sources) were captured. After eliminating 753 duplicates, 1,019 publications remained for more screening. The study selection process was done by two independent authors (CM and ZK). Based on initial screening, 1,002 records were excluded according to the titles and abstracts and 17 trials remained for full-text extraction. In the secondary screening, eight publications were excluded for the following reasons: combination with other components (*n* = 4) (McMahon et al., 2006; McNeill et al., 2007; Kennedy et al., 2011; Walker et al., 2012), conducted on the same population (*n* = 2) (Ma et al., 2016a,b), and trials with insufficient data (*n* = 2) (Durga et al., 2007; Farhana et al., 2016). In total, 9 trials with 10 effect sizes were included (Fioravanti et al., 1997; Sommer et al., 2003; Eussen et al., 2006; Pathansali et al., 2006; Connelly et al., 2008; Loria-Kohen et al., 2013; Chen et al., 2016; Ma et al., 2019; Bai et al., 2021). The general characteristics of these trials are shown in Figure 1.

### Study characteristics

The detailed characteristics of the nine included studies are summarized in Table 2. All trials had a randomized controlled parallel trial design. These trials were published between 1997 and 2021 and performed in Italy (Fioravanti et al., 1997), the United States (Sommer et al., 2003), the United Kingdom (Pathansali et al., 2006; Connelly et al., 2008), Spain (Loria-Kohen et al., 2013), Netherland (Eussen et al., 2006), and China (Chen et al., 2016; Ma et al., 2019; Bai et al., 2021). Overall, 641 participants including 326 subjects in the intervention group and 315 subjects in the control group participated in these trials. The age range of participants was from 22 to 83. The duration of intervention in these trials varied from 4 to 96 weeks, and the dosage of folic acid varied from 0.4 to 20 mg/d. All the articles were randomized controlled parallel trials and performed on both genders. Out of nine studies, two studies were conducted on patients with MCI (Ma et al., 2019; Bai et al., 2021), two trials on patients with AD (Connelly et al., 2008; Chen et al., 2016), one RCT on subjects with dementia and low normal folic acid levels (Sommer et al., 2003), one study on healthy elderly subjects (Pathansali et al., 2006), one trial on people aged >70 years with mild vitamin B-12 deficiency (Eussen et al., 2006) and one RCT on individuals with eating disorders (Loria-Kohen et al., 2013).

**TABLE 2:** General characteristics of randomized, double-blind, placebo-controlled parallel trial.

References	Country	Health status of subjects	Gender	Participants: Folic acid/Placebo	Duration (week)	Mean age (year)	Intervention		questionnaire	Outcomes
							treatment group	Placebo		
Fioravanti et al., 1997	Italy	Memory complaints in the past 2 years	Both	16/14	8	80.25	Folic acid (15 mg/d)	Placebo	Randt Memory Test (RMT)	Memory
Sommer et al., 2003	USA	Dementia and low-normal folic acid levels	Both	4/3	10	76.25	Folic acid (20 mg/d)	Placebo	Wechsler Memory Scale (WMS)	Memory
Pathansali et al., 2006	UK	Healthy elderly subjects	Both	12/12	4	72.3	Folic acid (5 mg/d)	Placebo	Scanning Memory Sets (SMS)	Memory
Eussen et al., 2006	Netherlands	Aged >70 years with mild vitamin B-12 deficiency	Both	51/54	24	83	Folic acid (0.4 mg/d)	Placebo	Wechsler Adult Intelligence Scale (WAIS)	Memory
Connelly et al., 2008	UK	Alzheimer’s disease	Both	23/18	24	75.65	Folic acid (1 mg/d)	Placebo	Mini-Mental State Examination (MMSE)	Memory
Loria-Kohen et al., 2013	Spain	Eating disorder	Both	14/10	24	22.3	Folic acid (10 mg/d)	Placebo	Stroop color-word interference test	Memory
Chen et al., 2016	China	Alzheimer’s disease	Both	61/60	24	68.1	Folic acid (1.25 mg/d)	Placebo	The Mini-Mental State Examination (MMSE)	Memory
Ma et al., 2016b	China	Mild Cognitive Impairment (MCI)	Both	76/75	96	74.8	Folic acid (0.4 mg/d)	Placebo	Wechsler Adult Intelligence Scale-Revised (WAIS-RC)	Memory
Bai et al., 2021	China	Mild Cognitive Impairment (MCI)	Both	35/33	48	67.51	Folic acid (0.8 mg/d)	Placebo	Wechsler Adult Intelligence Scale-Revised (WAIS-RC)	Memory
Bai et al., 2021	China	Mild Cognitive Impairment (MCI)	Both	34/36	48	66.74	Folic acid (0.8 mg/d)	Placebo	Wechsler Adult Intelligence Scale-Revised (WAIS-RC)	Memory

### Effect of folic supplementation on memory

**FIGURE 2:**
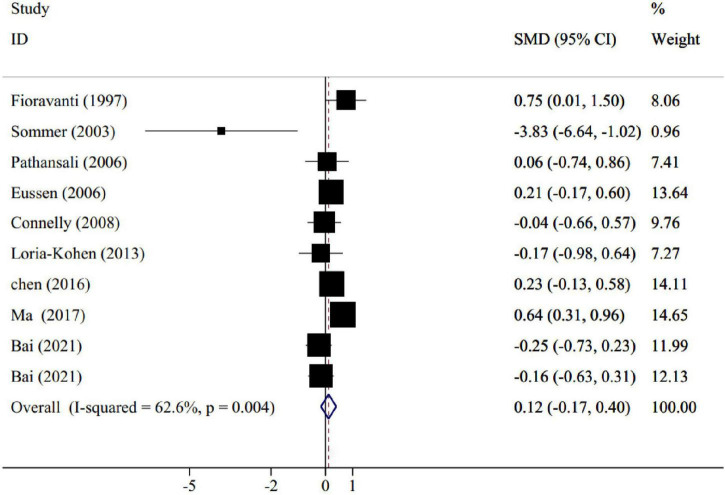
Forest plot for the effect of folic acid supplementation on memory, expressed as standardized mean differences between intervention and control groups. Weights are from random effects analysis.

The pooled results of nine trials [including 10 effect sizes; One study has got four groups [folic acid+docosahexaenoic acid (DHA), folic acid group, DHA group, and control group] we compared the folic acid and control groups once, and again compared the folic acid+DHA and DHA groups, so there are two effect sizes (Bai et al., 2021)], containing 641 participants (intervention = 326, control = 315), revealed that folic acid supplementation did not significantly change memory score compared to placebo (SMD: 0.12; 95% CI: −0.17, 0.40, *P* = 0.418), with a moderate degree of heterogeneity (*I*^2^ = 62.6%, *p* = 0.004) (Figure 2). To determine the source of heterogeneity, subgroup analyses were done. Age and participant’s health status were two sources of study heterogeneity. Subgroup analysis also indicated that memory score was significantly improved in trials that administered folic acid lower than 1 mg/d (SMD: 0.22; 95% CI: 0.02–0.42; *p* = 0.03). In addition, a significant improvement on memory score was observed in trials lasted ≥6 months of folic acid intervention (SMD: 0.18; 95% CI: 0.02–0.35; *p* = 0.02), conducted in eastern countries (SMD: 0.22; 95% CI: 0.03–0.42; *p* = 0.02), and in subjects ≥70 years (SMD: 0.37; 95% CI: 0.15–0.58; *p* = 0.01). The detailed results for subgroup analyses are summarized in Table 3.

**TABLE 3:** Pooled estimates of folic acid supplementation among different subgroups.

Group	Number of trials	SMD (95% CI)	P-effect	*I*^2^ (%)	*P*-within subgroup heterogeneity	*P*-between subgroup heterogeneity
Folic acid dosage						0.67
<1 mg/d	4	0.22 (0.02, 0.42)	0.03	75.9	0.01	
≥1 mg/d	6	0.15 (−0.10, 0.40)	0.23	56.3	0.04	
Duration						0.76
<6 months	3	0.27 (−0.25, 0.81)	0.31	80.2	0.01	
≥6 months	7	0.18 (0.02, 0.35)	0.02	56.8	0.03	
Study location						0.61
Western countries	6	0.14 (−0.12, 0.40)	0.30	56.1	0.04	
Eastern countries	4	0.22 (0.03, 0.42)	0.02	75.9	0.01	
Mean age						0.01
<70 years	4	−0.01 (−0.25, 0.21)	0.89	5.5	0.36	
≥70 years	6	0.37 (0.15, 0.58)	0.01	67.0	0.01	
Sample size						0.48
<50	5	0.07 (−0.28, 0.44)	0.67	64.1	0.02	
≥50	5	0.22 (0.04, 0.39)	0.01	67.9	0.01	
Health status						0.82
Mild Cognitive Impairment	3	0.22 (−0.007, 0.46)	0.06	83.9	0.01	
Alzheimer’s disease	3	0.11 (−0.19, 0.41)	0.48	75.7	0.01	
Other	4	0.22 (−0.06, 0.51)	0.13	1.0	0.34	

### Dose-response analysis

**FIGURE 3:**
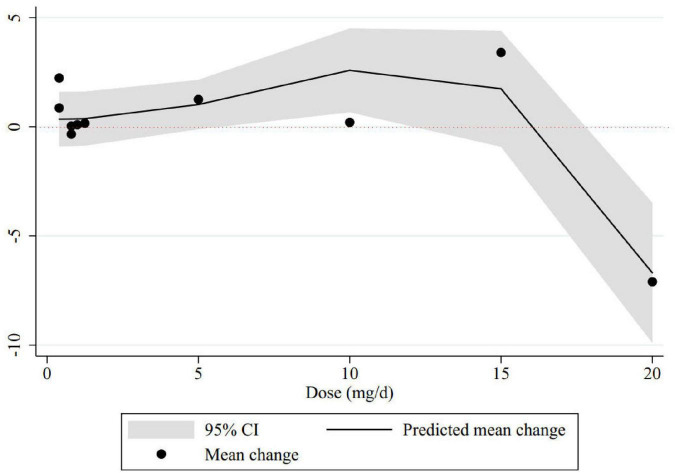
Non-linear dose-response relations between folic acid dosage (mg/d) and memory. The 95% CI is revealed in the shaded regions.

The non-linear dose-response analysis showed a significant non-linear association between folic acid dose and memory improvement (P_*non–linearity*_ = 0.01). Memory levels significantly increased with folic acid supplements at the dosage of 6–11 mg/day. Folic acid supplementation higher than 17 mg per day resulted in a significant decreasing trend in memory score (Figure 3).

### Sensitivity analysis and publication bias

**FIGURE 4:**
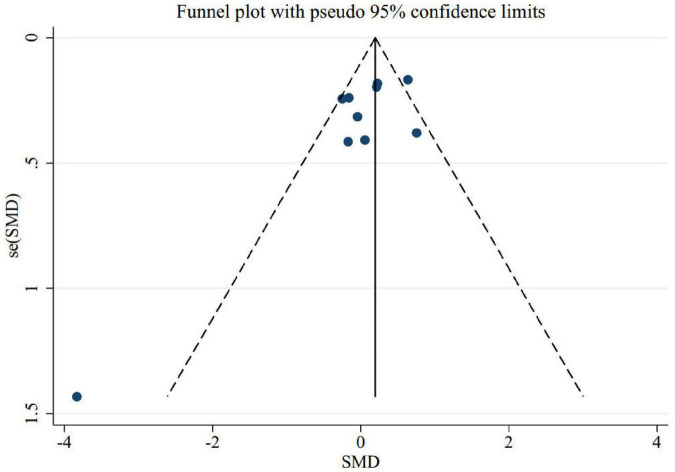
Funnel plot for the effect of folic acid supplementation on memory to identify publication bias.

To find out the effect of each trial on the pooled effect size, we excluded each study step-by-step from overall analysis. There were no significant effects of any individual trials on pooled effect size, according to our finding. The examination of publication bias by visual inspection of the funnel plot indicated asymmetry (Figure 4). However, Egger’s test showed no evidence of publication bias for trials evaluating the effect of folic acid supplementation on memory (*p* = 0.1).

### Grading the evidence

The certainty of evidence was rated using the GRADE approach. The certainty of evidence was rated moderate due to a downgrade for serious imprecision (Table 4).

**TABLE 4:** Grading of recommendations assessment, development and evaluation (GRADE) evidence table for the effect of folic acid supplementation on memory.

Certainty assessment							No of patients		Effect (95% CI)	Certainty	Importance
No of studies	Study design	Risk of bias	Inconsistency	Indirectness	Imprecision	Other considerations	Intervention	Control			
10	Randomized trials	Not serious	Not serious^a^	Not serious	Serious^b^	None	326	315	SMD 0.12 SD (95% CI: −0.17, 0.40)	ⴲⴲⴲ◯ Moderate	Important

CI, confidence interval; RR, relative risk; SD, standard deviation; SMD, standardized mean difference.

^a^serious inconsistency (*I*^2^ = 63%), explained by age and health status in the subgroup analyses. Not downgraded.

^b^serious imprecision since optimal information size was not met (*n* = 800). Downgraded.

## Discussion

This meta-analysis aimed to systematically evaluate the strength of current research on the efficacy of folic acid supplementation on memory improvement. The overall results revealed no benefits for folic acid supplementation over placebo in adult population on memory improvement. However, subgroup analysis showed that supplementation with folic acid had favorable effects on memory levels considering the following conditions: (1) doses lower than 1 mg/day, (2) treatment lasting more than 6 months, (3) conducted in eastern countries, and (4) in participants equal to or older than 70 years old. Moreover, dose-response analysis showed a significant increment in memory level at doses of 6–11 mg/d and a significant decrement at doses of 17–20 mg/d.

Folic acid is a group of heterocyclic compounds consisting of 4- (pterin-6-methylamino) benzoic acid and one or more L-glutamic acids. In addition, it is a co-factor in the process of one-carbon metabolism (OCM) that plays an important role in the flexibility of nerve cells and maintaining the integrity of neurons (Bailey and Gregory, 1999; Li et al., 2013). Aging causes a significant decrease in the concentration of folic acid and its constituent metabolite S-adenosylmethionine in cerebrospinal fluid and ultimately causes hyperhomocysteinemia (Reynolds, 1968). Hyperhomocysteinemia has been shown to occur in various neurological pathologies such as dementia and Alzheimer’s disease (Clarke et al., 1998; Seshadri et al., 2002). Folic acid deficiency and high homocysteine levels also endanger neurons, causing DNA damage and apoptosis in the hippocampus, which is responsible for memory (Kruman et al., 2000). Therefore, the effect of folic acid supplementation on memory has been considered.

The results of our meta-analysis suggested that folic acid supplementation had no effect on memory improvement.

Consistent with our results, in Wald et al. (2010) performed a meta-analysis of nine placebo-controlled randomized trials involving 2,835 participants and found no effect on cognitive function within 3 years. This previous meta- analysis investigated the effect of folic acid, with or without other B vitamins, on cognitive decline, whereas our study examined the effect of folic acid alone on memory, so several experimental studies were excluded from our review (Bryan et al., 2002; Lewerin et al., 2005; Stott et al., 2005; McMahon et al., 2006; Durga et al., 2007; McNeill et al., 2007). Wald et al. (2010) included only trials with at least 20 participants, aged 45 years or older, whereas our study included subjects older than 18 years. Also, four studies were published after the publication of the previous meta-analysis, which were included in our meta-analysis (Loria-Kohen et al., 2013; Chen et al., 2016; Ma et al., 2016a; Bai et al., 2021). Other previous studies were systematic reviews, and such reviews were largely descriptive and lacked the power to provide a brief effect of folic acid supplementation on brain function (Balk et al., 2007). Meanwhile, observational studies have found an association between low serum folic acid levels and neocortical atrophy of the brain in people who died of Alzheimer’s disease (Snowdon et al., 2000). But it is not clear whether folic acid supplementation reduces the incidence of Alzheimer’s disease or not.

Folic acid plays a key role in stabilizing short-term and long-term memory and attenuating memory disorders (Reynolds, 2002; Enderami et al., 2018). However, the acceptable biochemical mechanism is not completely clear. Most of the available evidence points to the major role of folic acid-mediated one-carbon metabolism and DNA methylation events (Scott and Weir, 1998). In fact, folic acid increases the methylation potential and activity of DNA methyl transferases (DNMTs), modifies DNA methylation, and ultimately reduces β-amyloid precursor protein (APP) and Aβ protein levels, and can improve memory and cognition (Smith et al., 2012; Duncan et al., 2013). In addition, folic acid has antioxidant properties that counteract the effects of Alzheimer’s disease and other cognitive impairments (Archibald et al., 2013). Folic acid also inhibits tau phosphorylation and subsequent formation of neurofibrillary tangle by indirectly regulating cyclin-dependent protein phosphatase and glycogen synthase kinase activity (Zhang et al., 2009; Sontag and Sontag, 2014).

Also, our results showed that in doses lower than 1 mg per day, folic acid supplementation has a beneficial effect on memory, while this effect was not seen in higher doses. To prevent adverse health effects, the tolerable upper intake level (UL) of 1 mg per day was set as folic acid. Taking more than UL of folic acid can mask pernicious anemia, which causes neurological disorders caused by vitamin B12 deficiency (Scott and Weir, 1998; Sommer et al., 2003; Smith, 2008; Zhang et al., 2009; Smith et al., 2012; Archibald et al., 2013; Duncan et al., 2013; Ebara, 2017; Enderami et al., 2018). Also, high levels of folic acid can decrease the activity of the Na+, K+-ATPase enzyme. The proper functioning of this enzyme is related to the memory and learning process, because this enzyme continuously interacts with N-methyl-D-aspartate (NMDA) receptor which is located in the synaptic regions and it has an important role in the memory process (Vieira Carletti et al., 2016; Deniz et al., 2018).

There are several plausible explanations for our results. The duration of treatment in some trials might be too short (Sommer et al., 2003). As observed in the subgroup analysis, studies with interventions longer than 6 months showed that folic acid supplementation had a significant effect on improving memory. In addition, it is difficult to distinguish significant differences between groups as cognition is unlikely to decrease significantly, especially in healthy elderly, in this short period of time. People with high total plasma homocysteine (tHcy) may also benefit more by reducing homocysteine with folic acid supplementation (Flicker et al., 2006). Due to the lack of reported data on tHcy in the studies, we were unable to take these observations into account in the analysis. Another limitation of this study is the lack of reporting of dietary pattern in the included studies, so we could not consider this data in the analysis. Moreover, most of the studies were from Europe, Asia, and North America and limited data from Africa and South America. It may be necessary to consider the regional effect. In our study the heterogeneity was large so, we did subgroup analysis to identify the source of heterogeneity. It revealed that heterogeneity decreased for sample size, dosage, and health status.

As far as we know, this is the most comprehensive systematic review and meta-analysis to examine the effect of folic acid supplementation on memory. In addition, dose-response analysis was another strength of this review. Also, non-linear dose-response analysis showed a significant non-linear relationship between folic acid dosage and memory improvement. As the number of RCTs and sample size were very small, more studies are needed to support the results.

## Conclusion

This meta-analysis revealed that the most effect of folic acid supplementation on memory score was observed when administered at doses lower than 1 mg/day, long-term duration, and in subjects with 70 years and older. In addition, there was a non-linear relationship between folic acid dosage and memory improvement. Due to the small number of RCTs and sample size, further research is needed to support our results.

## Data availability statement

The original contributions presented in this study are included in the article/Supplementary material, further inquiries can be directed to the corresponding authors.

## Author contributions

ZV was the guarantor. CA, ZK, and SE wrote the manuscript. MG and HR conducted the literature search and performed the data extraction and quality assessment. HI and CA developed the search strategy. CA and AB conceived the study and performed the statistical analysis. All authors read and approved the final protocol manuscript.
