# Supplementary material for: Does folic acid supplementation have a positive effect on improving memory? A systematic review and meta-analysis of randomized controlled trials
Source: Front Aging Neurosci. 2022 Nov 28;14:966933. doi: 10.3389/fnagi.2022.966933 (PMC9742231; doi:10.3389/fnagi.2022.966933)
Supplement: Supplementary file 1 [file Table_1.DOCX]

**Supplementary Material**

**Does Folic Acid Supplementation Have a Positive Effect on Improving Memory? A systematic review and meta-analysis of randomized controlled trials**

**Supplemental Table 1: Search strategy used for MEDLINE (via PubMed and googlescholar)**

#1 (“Cognitions” OR “Dementia” OR “Alzheimer disease” OR Cognitive Function” OR “Cognitive Functions” OR Memory OR “cognitive performance” OR “cognitive decline” OR “cognitive impairment” OR “memory impairment” OR “Dementias” OR “Amentia” OR “Amentias” OR “Familial Dementia” OR “Familial Dementias” OR “Alzheimer Dementia” OR “Alzheimer Dementias” OR “Alzheimer's Disease” OR “Senile Dementia” OR “Alzheimer Type Dementia” OR “Alzheimer-Type Dementia” OR “Alzheimer Type Dementia” OR “Alzheimer Type Senile Dementia” OR “Primary Senile Degenerative Dementia” OR “Alzheimer Sclerosis” OR “Alzheimer Syndrome” OR “Alzheimer's Diseases” OR “Alzheimers Diseases” OR “Presenile Dementia” OR “Late Onset Alzheimer Disease” OR “Familial Alzheimer Disease” OR “Familial Alzheimer Diseases” OR “Early Onset Alzheimer Disease” OR “Presenile Alzheimer Dementia”)

#2 ("Folic acid" OR "Vitamin M" OR “Vitamin B9" OR “Pteroylglutamic Acid" OR “Folvite” OR “Folacin” OR “Folate” OR “Folates” OR “Tetrahydrofolates” OR “Formyltetrahydrofolates")

#3 (("Random Allocation" OR "Single-Blind Method" OR "Double-Blind Method" OR "Cross-Over Studies" OR "Clinical Trials as Topic" OR RCT OR "Intervention Studies" OR "intervention" OR "controlled trial" OR "randomized" OR "randomized" OR "random" OR "randomly" OR "placebo" OR "assignment" OR "Cross-Over").

#1 AND #2 AND #3

**Supplemental Table 2**: Results of risk of bias assessment for randomized clinical trials included in the current meta-analysis on the effects of Folic acid supplementation on memory.

| Study | Random sequence generation | Allocation concealment | Selective reporting | Blinding (participants and personnel) | Blinding (outcome assessment) | Incomplete outcome data | other source of bias | Overall quality |
| --- | --- | --- | --- | --- | --- | --- | --- | --- |
| Fioravanti (1997) ( | L | U | L | L | U | L | L | fair |
| Sommer (2003) | L | U | L | L | U | L | L | fair |
| Pathansali (2006) | L | L | L | L | U | L | L | fair |
| Eussen (2006) | L | L | L | L | U | L | L | fair |
| Connelly (2008) | L | L | L | L | L | L | L | good |
| Loria-Kohen (2013) | L | U | L | L | U | H | H | poor |
| chen (2016) | L | L | L | H | H | L | L | poor |
| Ma (2017) | L | L | L | L | U | L | L | good |
| Bai (2021) | L | L | L | L | L | L | L | good |

U; unclear risk of bias, L; low risk of bias, H; high risk of bias
